# 4,4′-(Anthracene-9,10-diylbis(ethyne-2,1-diyl))bis(1-methyl-1-pyridinium) Lead Iodide C_30_H_22_N_2_Pb_2_I_6_: A Highly Luminescent, Chemically and Thermally Stable One-Dimensional Hybrid Iodoplumbate

**DOI:** 10.1021/acs.chemmater.2c03798

**Published:** 2023-02-15

**Authors:** Lorenza Romagnoli, Andrea D’Annibale, Elena Blundo, Atanu Patra, Antonio Polimeni, Daniele Meggiolaro, Iryna Andrusenko, Danilo Marchetti, Mauro Gemmi, Alessandro Latini

**Affiliations:** † Dipartimento di Chimica, Sapienza Università di Roma, Piazzale Aldo Moro 5, Roma 00185, Italy; ‡ Dipartimento di Fisica, Sapienza Università di Roma, Piazzale Aldo Moro 5, Roma 00185, Italy; § Computational Laboratory for Hybrid/Organic Photovoltaics (CLHYO), Istituto CNR di Scienze e Tecnologie Chimiche “Giulio Natta” (CNR-SCITEC), Via Elce di Sotto 8, Perugia 06123, Italy; ∥ Electron Crystallography, Center for Materials Interfaces, Istituto Italiano di Tecnologia, Viale Rinaldo Piaggio 34, Pontedera 56025, Italy; ⊥ Department of Chemistry, Life Sciences and Environmental Sustainability, University of Parma, Parco Area delle Scienze 17/A, Parma (PR) 43124, Italy

## Abstract

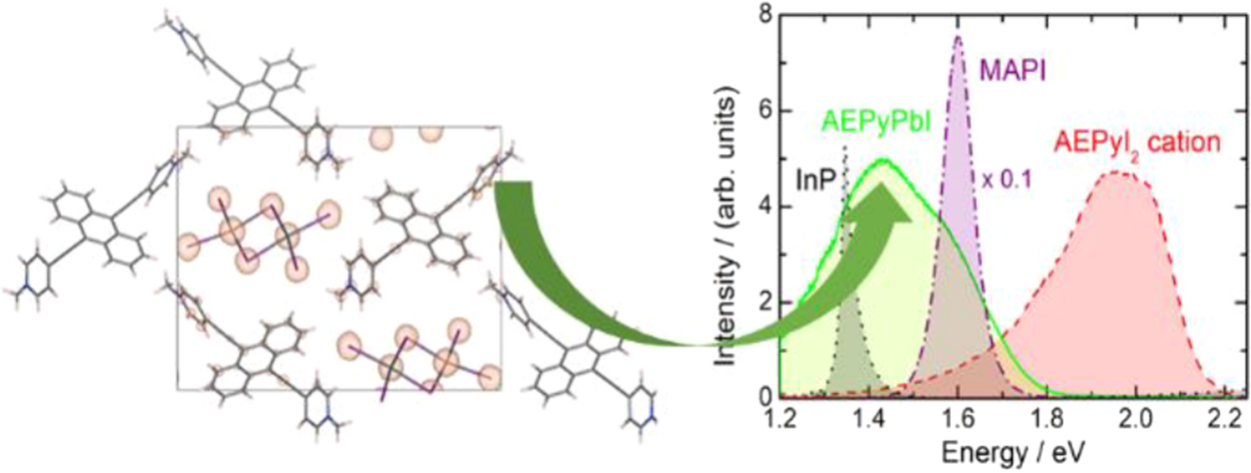

A new one-dimensional hybrid iodoplumbate, namely, 4,4′-(anthracene-9,10-diylbis(ethyne-2,1-diyl))bis(1-methyl-1-pyridinium) lead iodide C_30_H_22_N_2_Pb_2_I_6_ (AEPyPbI), is reported here for the first time with its complete characterization. The material exhibits remarkable thermal stability (up to 300 °C), and it is unreactive under ambient conditions toward water and atmospheric oxygen, due to the quaternary nature of the nitrogen atoms present in the organic cation. The cation exhibits strong visible fluorescence under ultraviolet (UV) irradiation, and when its iodide is combined with PbI_2_, it forms AEPyPb_2_I_6_, an efficient light-emitting material, with a photoluminescence emission intensity comparable to that of high-quality InP epilayers. The structure determination was obtained using three-dimensional electron diffraction, and the material was extensively studied by using a wide range of techniques, such as X-ray powder diffraction, diffuse reflectance UV–visible spectroscopy, thermogravimetry-differential thermal analysis, elemental analysis, Raman and infrared spectroscopies, and photoluminescence spectroscopy. The emissive properties of the material were correlated with its electronic structure by using state-of-the-art theoretical calculations. The complex, highly conjugated electronic structure of the cation interacts strongly with that of the Pb–I network, giving rise to the peculiar optoelectronic properties of AEPyPb_2_I_6_. The material, considering its relatively easy synthesis and stability, shows promise for light-emitting and photovoltaic devices. The use of highly conjugated quaternary ammonium cations may be useful for the development of new hybrid iodoplumbates and perovskites with optoelectronic properties tailored for specific applications.

## Introduction

Metal halide hybrid perovskites (MHHPs) are very intriguing semiconductors because of their excellent optoelectronic properties, which can be varied by simple chemical modifications of the material composition.^1^ The relatively low cost of production and the ease of synthesis^1,2^ are particularly advantageous for the large-scale diffusion of these materials in several fields of optoelectronics, including photovoltaics,^3^ photodetectors,^4^ and light-emitting devices.^5,6^

Despite these encouraging premises and the enormous amount of research work done worldwide on these materials, the implementation of MHHPs in commercial devices is mostly limited by their long-term instability, particularly in the “hottest” applications, such as photovoltaic devices.^7,8^

One of the factors that is strongly reducing the long-term stability of the MHHP bearing organic cations is the relatively low stability of the organics toward acid–base reactions.^9−12^ As an example, for methylammonium lead iodide CH_3_NH_3_PbI_3_, both its water sensitivity and thermal instability stem from the Brønsted acidity of the methylammonium cation:^9^
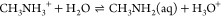

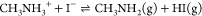


The use of organoammonium cations with lower Brønsted acidity greatly enhances the chemical and thermal stability of the corresponding MHHPs.^13−15^

Depending on the spatial configuration of the Pb–I network, MHHPs may be classified according to their dimensionality; i.e., in three-dimensional (3D) perovskites, the PbI_6_ octahedra are three-dimensionally connected by sharing all their vertices, in 2D, the PbI_6_ octahedra are connected to form sheets, in 1D, to form chains, and in 0D, to form isolated Pb–I polyhedra. The growth of MHHPs with a specific dimensionality may be carried out by a judicious choice of cation size.^16^

The deriving electronic properties in the case of “silent” organic cations (i.e., cations whose electronic structure does not interact significantly with the electronic structure of the inorganic network) are intimately connected to the thickness of the inorganic sheets and the discontinuity of the dielectric constant between the inorganic and the organic components of the lattice,^17^ modulating the quantum and dielectric confinement of the inorganics, respectively. In low dimensional perovskites, both these effects lead to higher band gaps and exciton binding energies compared to 3D analogues.^16,17^

MHHPs containing highly conjugated cations like viologens show additional optoelectronic properties due to the interactions between the electronic structures of the organic cations and those of the inorganic network.^15,18−25^ Our previous work on phenylviologen lead iodide,^15,26^ which was demonstrated to be very stable both chemically and thermally and possessing interesting light-emitting properties, stimulated our interest toward new hybrid metal halide perovskites and, generally speaking, iodoplumbates and other halometallates with highly conjugated cations and quaternarized nitrogen atoms.

In the present work, we present the synthesis and the complete characterization of a new 1D iodoplumbate, namely, 4,4′-(anthracene-9,10-diylbis(ethyne-2,1-diyl))bis(1-methyl-1-pyridinium) lead iodide C_30_H_22_N_2_Pb_2_I_6_ (AEPyPbI). The thorough experimental characterization is supported by state-of-the-art calculations, providing insights into the basic properties of the material. AEPyPbI displays remarkable thermal stability (up to 300 °C) and insensitivity to water, due to the quaternary nature of the organic ammonium cations, and displays exceptionally high photoluminescence (PL) yield, comparable to that of high-quality InP epilayers.

The synthesis of the cation is relatively easy, being based on the well-established and highly effective Sonogashira coupling reaction, and the perovskite can be simply obtained by mixing equimolar solutions of the organic cation iodide and lead iodide at room temperature with no special precautions. The cation was successfully used also for the synthesis of another highly luminescent iodometallate, 4,4′-(anthracene-9,10-diylbis(ethyne-2,1-diyl))bis(1-methyl-1-pyridinium) bismuth iodide.^27^ The material’s easy preparation and intriguing optoelectronic properties render it a promising candidate for light-emitting and photovoltaic devices. Additionally, the use of highly conjugated quaternary ammonium organic cations opens up new perspectives for the design of MHHPs with specifically tailored properties.

## Results and Discussion

**Figure 1:**
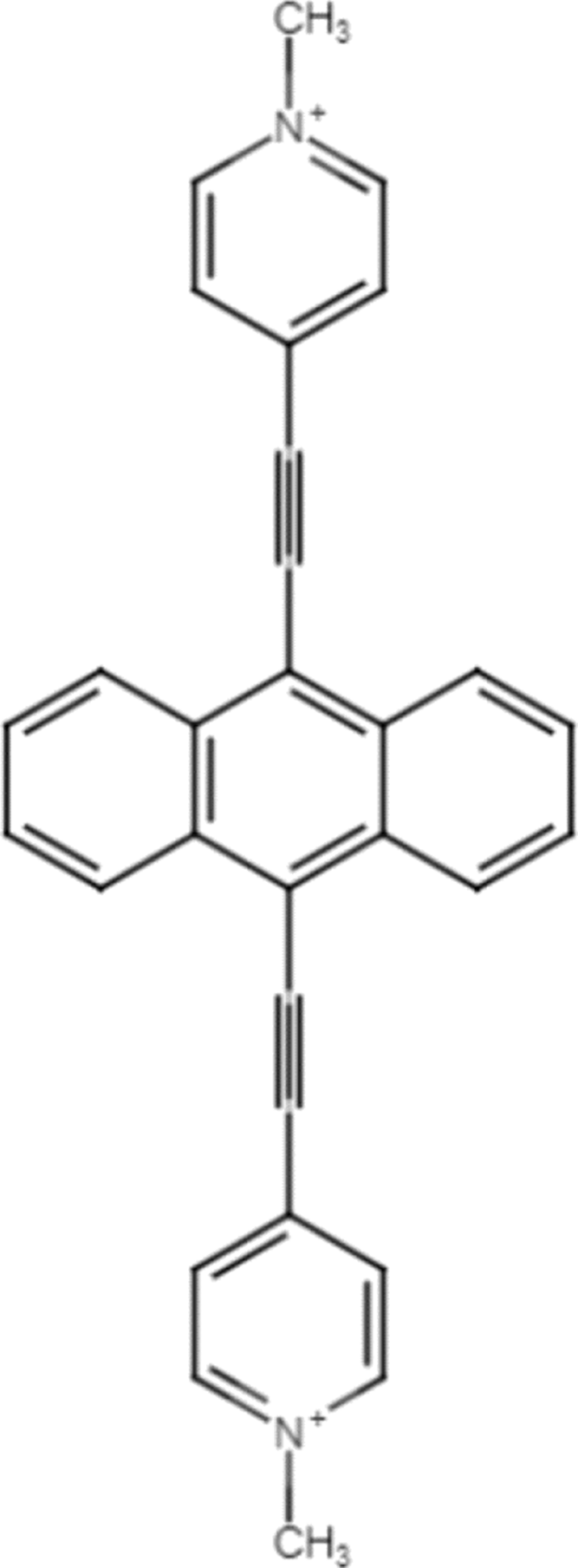
Structure of the AEPy^2+^ cation.

The structure of the AEPy^2+^ cation is shown in Figure 1.

**Figure 2:**
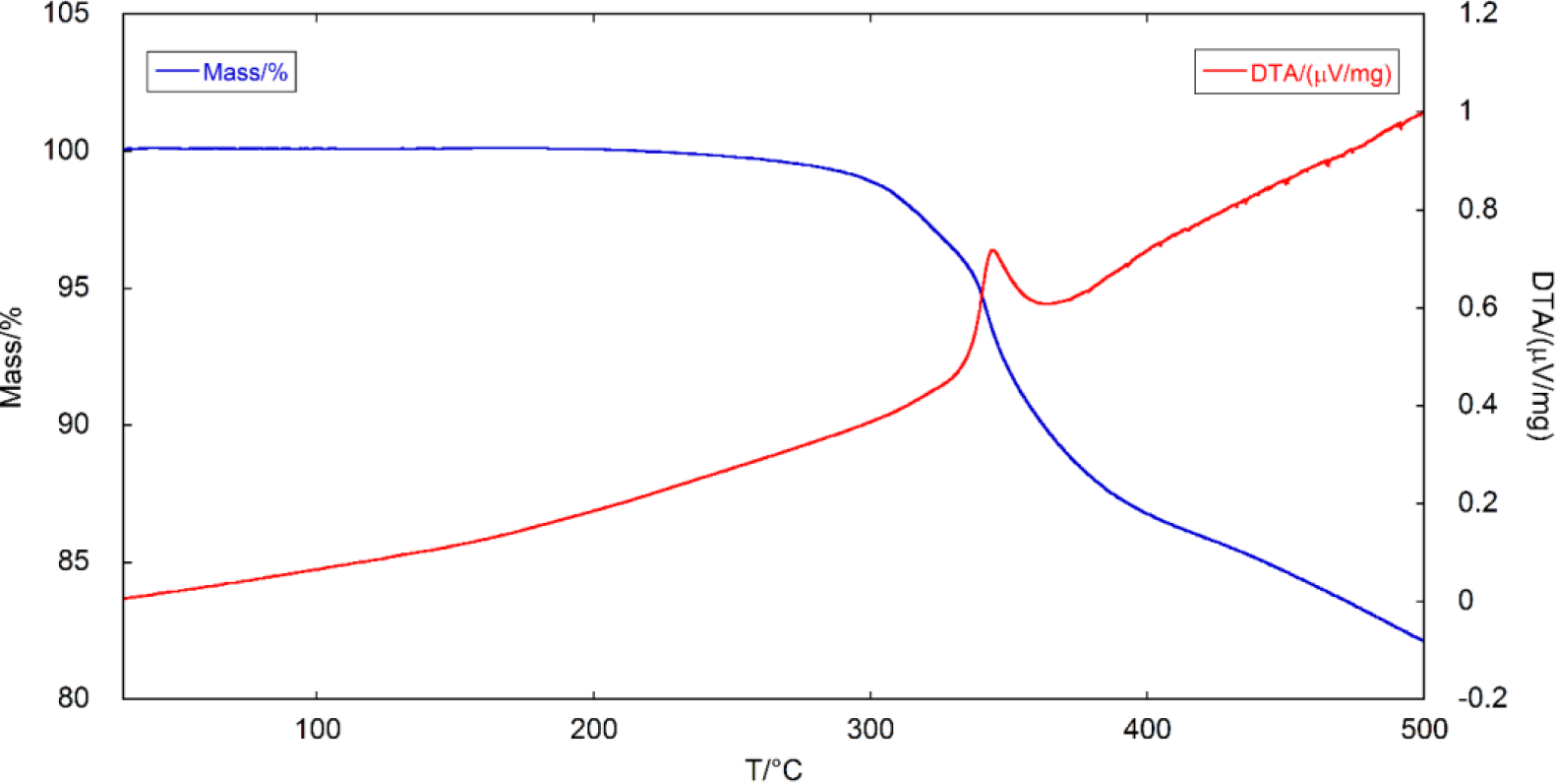
TG-DTA profiles of AEPyPbI.

AEPyPbI was synthesized as a crystalline, air and moisture-stable black powder by reacting an aqueous solution of AEPyI_2_ with a solution containing an equimolar amount of PbI_2_ and an excess of NaI in acetone (see the Supporting Information). AEPyPbI is fairly soluble in *N*,*N*-dimethylformamide (DMF, ∼100 mg in 80 mL at room temperature). The material displays remarkable thermal stability, decomposing endothermally around 300 °C as demonstrated by the thermogravimetry-differential thermal analysis (TG-DTA), see Figure 2.

The composition of AEPyPbI was verified by determining the total carbon content, which was found to be 22.358% vs a calculated value of 22.714%, in excellent agreement.

The electrospray ionization mass spectrometry (ESI-MS) spectrum of AEPyPbI dissolved in methanol (in which the compound is very slightly soluble, Figures S1 and S2 of the Supporting Information) confirms the identity of the cationic and anionic species. The positive ions mass spectrum has two prominent peaks at *m*/*z* 205 and 395, attributable to AEPy^2+^ and the monopositive species due to AEPy^2+^ that lost a CH_3_^+^ fragment, respectively. The three intense peaks in the negative ions mass spectrum at *m*/*z* 127, 381, and 588 can be attributed to I^–^, I_3_^–^, and Pb_2_I_6_^2–^, respectively.

While the synthesis of AEPyPbI was relatively simple, the growth of single crystals suitable for crystal structure determination by X-ray diffraction has been a true challenge and unfortunately no technique among those tried gave positive results. Trials were made with antisolvent vapor diffusion using different combinations of solvent and antisolvent; other trials were made by stratifying a layer of an antisolvent over a solution of AEPyPbI; finally, hydrothermal methods were also tried. With no suitable single crystals for X-ray diffraction, the structure could be solved only by powder methods or by electron diffraction (ED). The use of powder methods, considering the high number of electrons of Pb and I atoms, was deemed neutron techniques, but to acquire high-quality powder patterns totally deuterated samples were necessary, making the approach impractical.

The difficulty in obtaining relatively large single crystals is probably due to the polymeric nature of the inorganic lattice that, coupled with the bulky cations, makes the packing of the crystal difficult.

It is common for the crystal size to be in the submicrometric range, making standard crystallographic investigations for structure solutions a challenge. For such crystals, X-ray powder diffraction (XRPD) is often the only possible option. However, structure solution from XRPD may fail in case of large unit cells, low symmetry space groups, strong peak broadening, and also for the presence of possible contaminants.^28^ Furthermore, for hybrid organic–inorganic compounds, the presence of heavy atoms may lead to powder diffraction patterns with a large contribution of the inorganic fraction, which in some cases can totally masks the organic fraction scattering contribution.^29^

On the other hand, if the phase of interest appears as submicrometric grains, 3D electron diffraction (3D ED) has proved to be a reliable method for structure determination^30^ and has found wide application in the case of hybrid structures.^31^ To fully exploit the potentialities of both XRPD and 3D ED, the following protocol has been adopted: (i) any new synthesis is characterized by XRPD; (ii) if the XRPD pattern cannot be fully indexed with known structures, 3D ED data are collected from few single nanocrystals to identify the new phase present in the sample and to measure the related unit cell; and (iii) if an unknown phase is detected, its crystal structure is solved by 3D ED and refined by Rietveld refinement against XRPD. Such a combination of methods was already successfully used in several pure organic compounds^32−35^ and hybrid organic–inorganic materials.^29,36−38^

**Figure 3:**
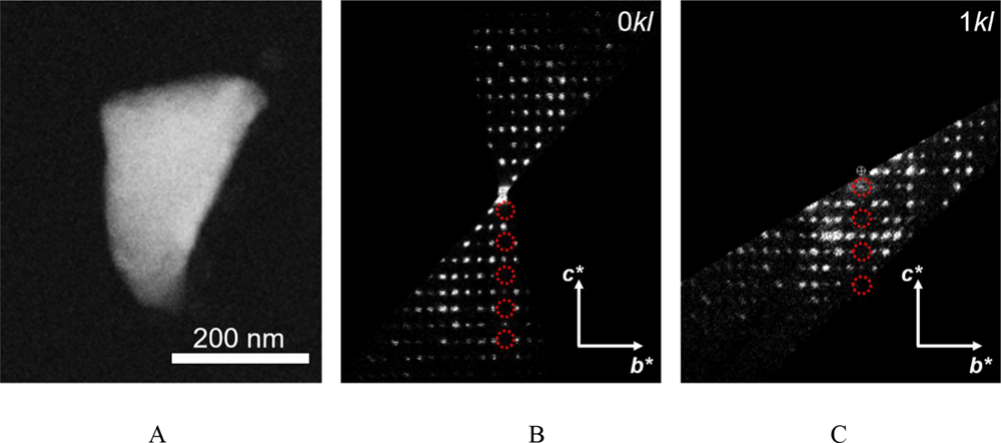
(A) High angle annular dark field STEM (HAADF-STEM) image of the AEPyPbI single crystal fragment used for 3D ED data collection. (B and C) Selected planar cuts of the 3D reciprocal space reconstruction of AEPyPbI, revealing extinction conditions *h*0*l*:*l* = 2*n* (highlighted in red). *The displayed planar cuts come from the two different 3D ED datasets that were selected for the integration of reflection intensities.

3D ED data were recorded from several crystal fragments with sizes less than 1 μm (Figure 3A). All datasets consistently showed the same primitive monoclinic unit cell. Among those, the two displaying the largest angular tilt range and devoid of artifacts induced by polycrystallinity were selected for the structure determination. The approximate cell parameters obtained from 3D ED are *a* = 4.7, *b* = 23.0, *c* = 17.8 Å, and β = 105.0°. The cell parameters were further refined and validated with a Le Bail fitting against XRPD, from which the following values were obtained: *a* = 4.6628(5), *b* = 22.902(4), *c* = 17.696(4) Å, and β = 105.3477(3)°. Reciprocal space sections on 3D ED data revealed *h*0*l*:*l* = 2*n* as extinction condition (Figure 3B,C), pointing convincingly to a monoclinic space group *Pc* (7) or *P*2/*c* (13). Reflection intensities extracted from both datasets were merged according to the observed Laue symmetry and by a scale factor derived from the comparison of the strongest common reflections.

The structure solution was performed in two steps. First, Pb–I inorganic clusters were obtained by standard direct methods (SDMs) that resulted in the automatic localization of two Pb and six I atoms in the asymmetric unit. Second, two independent ligand molecules were added by simulated annealing (SA). The molecular model was deduced from structure CCDC 2181402 containing the same cation and modeled as a unique fragment. No anti-bump restraint was used. This method was applied successfully to 3D ED data for the determination of a covalent organic framework^39^ and important pharmaceuticals.^40,41^ Structure solution attempts in space group *P*2/*c* (13) proved unstable and could not be refined. The best crystallographic model was obtained in space group *Pc* (7) and farther kinematically least-squares refined against 3D ED data, after imposing constraints on the aromatic rings and the assignment of all hydrogen atoms to calculated positions. Additionally, restraints were imposed on Pb–I interatomic distances and the planarity of the flat blocks that make up the molecule. More details about structure determination and refinement are reported in Table 1.

**Table 1:** Selected Parameters from Structure Solution (*SIR2014*) and Refinement (*SHELXL*) Based on the 3D ED Data

crystallographic information	
asymmetric unit content	C_30_H_22_N_2_Pb_2_I_6_
*Z*	2
space group	*Pc* (7)
*a* (Å)	4.6628(5)
*b* (Å)	22.902(4)
*c* (Å)	17.696(4)
β (°)	105.3477(3)
volume (Å^3^)	1823.804(6)
structure solution parameters (*SIR2014*)	
data resolution (Å)	0.9
no. of sampled reflections	5392
no. of independent reflections	2180
independent reflection coverage (%)	81
global thermal factor *U*_iso_ (Å^2^)	0.03045
*R*_int_(*F*) (%)	18.95
CF (%)	36.371
structure refinement parameters (*SHELXL*)	
data resolution (Å)	0.9
*R*_int_(*F*2) (%)	30.87
no. of reflections (all)	5219
no. of reflections (>4σ)	871
*R*1(4σ) (%)	34.50
goodness-of-fit	1.334
structure refined parameters—PXRD (JANA 2006)	
data resolution (Å)	1.09
*R*_p_	3.50
w*R*_p_	5.32
no. of reflections (all)	2987
no. of reflections (>3σ)	2819
*R*(3σ) (%)	17.41
w*R*(3σ) (%)	18.49

The high *R*-value of the kinematical refinement can be expected due to the unavoidable presence of dynamical scattering, which is higher in the case of the presence of heavy atoms, and due to imperfections in the data collection related to the small crystal size and to the beam movement over different positions of the same crystal in order to reduce the beam damage.

**Figure 4:**
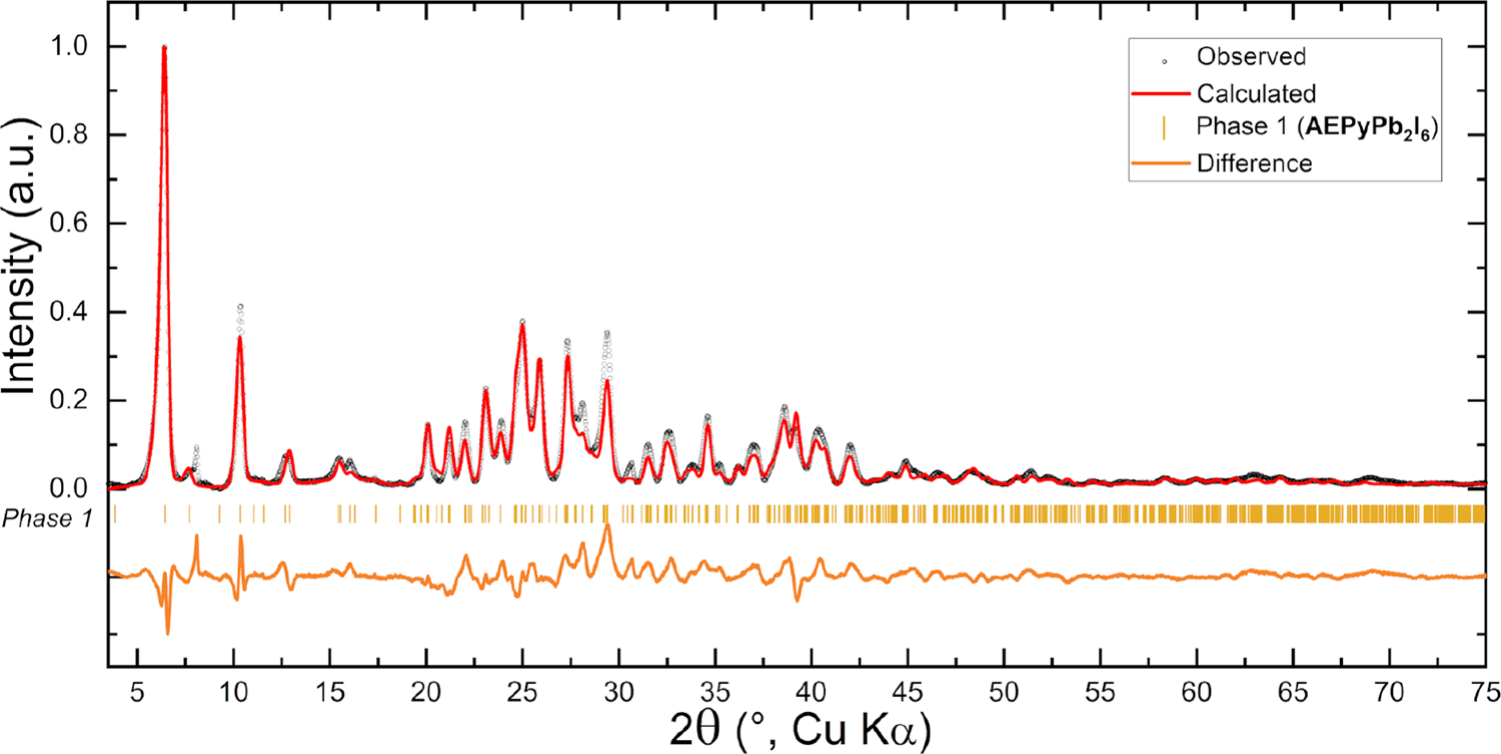
Final profile fit obtained by a Rietveld refinement on AEPyPbI, performed starting from the structural model obtained by 3D ED.

**Figure 5:**
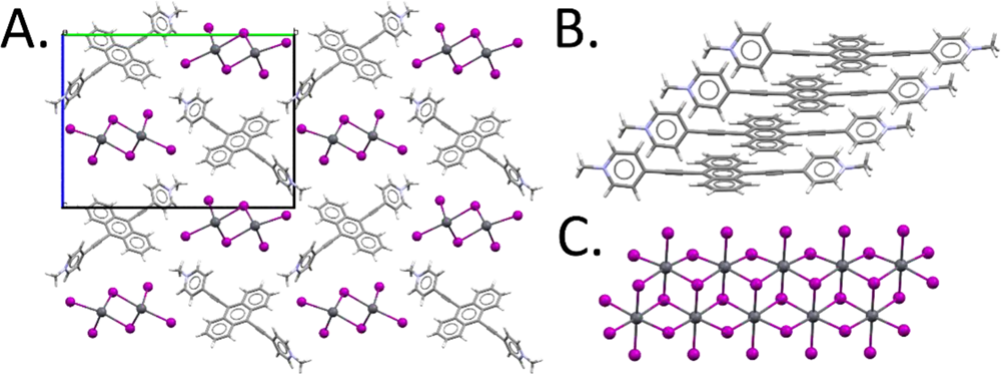
(A) Crystal structure of AEPyPbI oriented along the *a* axis. The almost centered packing of Pb_2_I_6_ chains is clearly visible. (B) Representation of AEPy molecular packing. (C) Representation of the Pb_2_I_6_ double chain. Color code: Pb (dark-gray), I (purple), N (blue), C (light-gray), and H (white).

The obtained 3D ED structural model was subsequently improved by Rietveld refinement on XRPD data (Figure 4). The refinement converged to *R*_wp_ = 5.32, *R*_F_(obs) = 17.41, and w*R*_F_(obs) = 18.49% without any significant modification (Figure 4). The final structure model is shown in Figure 5A. The AEPyPbI crystal structure is made of well-separated Pb_2_I_6_ double chains of edge-sharing octahedra, which runs along *a* direction that in the *b*–*c* plane are arranged in an almost centered lattice leaving room for the AEPy molecules, which piles up in the same *a* direction through π–π interactions (Figure 5B,C).

**Figure 6:**
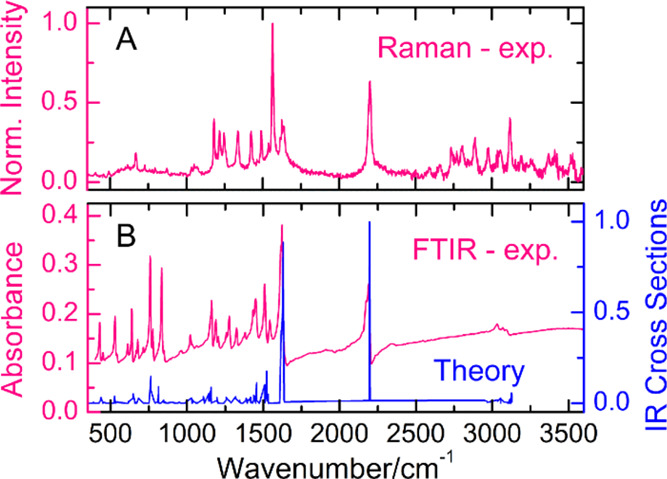
Raman (panel A) and FTIR (panel B) spectra of AEPyPbI. The latter is compared to the IR spectrum calculated by DFPT.

The vibrational properties of AEPyPbI were investigated by Raman and Fourier transform infrared (FTIR) spectroscopies. In Figure 6, the experimental Raman and FTIR spectra are compared with the simulated IR spectrum, obtained by density functional perturbation theory (DFPT) calculations^42^ (see Computational Details). Overall, a good agreement between experiment and theory is observed. AEPyPbI shows vibrational features deriving from the oscillations of the different components of the perovskite. The low-energy portion of the spectrum, below 100 cm^–1^, is dominated by the bending and stretching of the inorganics; modes between 100–200 cm^–1^ are associated with the torsion of the organics within the 1D channel of the material; the frequency region between 200–600 cm^–1^ is dominated by the bending of the organic cations; beyond 600 cm^–1^ only the stretching modes of the cation are present. The most intense peaks at ∼1600 and ∼2200 cm^–1^ are associated with the stretching of the carbon ring and triple C–C bond, respectively.

**Figure 7:**
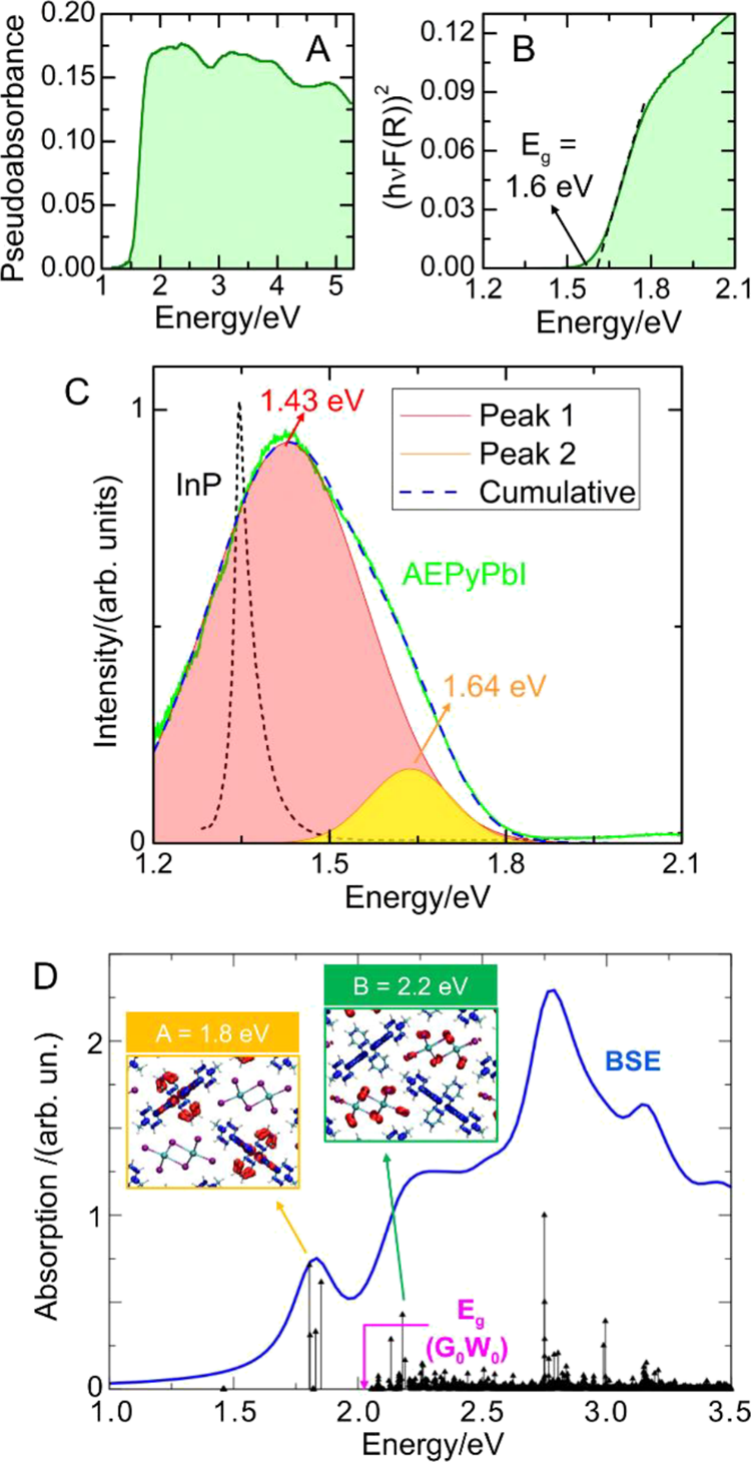
(A) UV–vis spectrum of AEPyPbI. (B) Tauc’s plot (assuming a direct optical transition) for the determination of the band gap value *E*_g_. (C) Photoluminescence (PL) spectrum of AEPyPbI (light green) and corresponding fit (blue dashed line) with two Gaussian functions (pink and orange). The PL spectrum of a high-quality InP epilayer (black short-dashed line) acquired under analogous conditions is also shown for comparison, demonstrating the high emission efficiency of AEPyPbI. (D) Simulated optical spectrum of AEPyPbI obtained by solving the BS equation on top of the *G*_0_*W*_0_ calculation. The most intense exciton peaks at lower energies are highlighted in orange and green. Insets: main Kohn–Sham states involved in the two transitions, along with the relative transition energies (hole = red; electron = blue).

Finally, we investigated the optical and electronic properties of AEPyPbI by a series of experiments and theoretical characterizations. The experimental absorption spectrum is shown in Figure 7A. Figure 7B shows instead the corresponding Tauc’s plot, obtained by assuming a direct optical transition. From Tauc’s plot, an optical bandgap of 1.6 eV is estimated.

The emission efficiency of the material was investigated by PL spectroscopy measurements performed on the AEPyPbI powder. Figure 7C shows the PL spectrum of AEPyPbI (light green). The spectrum was acquired with a Si charge-coupled device (CCD), and the system response was duly taken into account. Since the system response drops at ∼1.2 eV, the spectrum is shown only until that energy value. To verify that no lower energy states were present, the PL signal was measured also by an InGaAs linear array, as shown in Supporting Figure S5. The spectrum in Figure 7C can be well fitted by two Gaussians, centered at 1.43 eV (red) and 1.64 eV (orange). The PL emission of the perovskite is indeed high, as revealed by the comparison with the PL signal of a high-quality 3 micron-thick (100)InP epilayer grown by metal–organic chemical vapor deposition at a temperature of 650 °C (black dashed line). AEPyPbI and the InP epilayer show similar peak intensities, while the integrated area of the PL signal of AEPyPbI is about one order of magnitude larger than that of InP. To get further quantitative information on the PL intensity of AEPyPbI, in Supporting Figure S6, we compare it also with the PL signal of a CH_3_NH_3_PbI_3_ (MAPI) perovskite. The integrated area of the PL signal of MAPI is about 3.1 times greater than that of AEPyPbI, confirming a high PL efficiency for the latter. The PL quantum yield^43−45^ of AEPyPbI was also measured and compared to those of InP and MAPI, see the discussion of Supporting Figure S6. In the same figure, we also show the PL signal of the AEPyI_2_ cation, showing how its PL signal is bright and it is centered at much higher energy (∼1.97 eV) than that of the AEPyPbI perovskite.

To test the robustness of the perovskite upon photoexcitation, we performed PL measurements by varying the excitation power from 44 nW to 900 μW, focused through a 20× objective with NA = 0.4. As shown in Supporting Figure S7, no line shape variations are observed until 200 μW, which testifies the robustness of this perovskite against photoexcitation. At higher powers, a broadening is observed, followed by a quenching of the PL intensity.

The nature of optical excitations in the AEPyPbI perovskite was correlated to its electronic structure by means of DFT calculations. The electronic band structure and the projected density of states (PDOS) of AEPyPbI were calculated by using the PBE functional^46^ and including spin–orbit coupling (SOC), see Figure S7 of the SI. At this level of theory, the perovskite shows an indirect band gap of 0.71 eV. The analysis of the PDOS indicates that the VBM is mainly derived by I and cation orbitals, while the conduction band minimum (CBM) is entirely derived by the cation orbitals, in agreement with the localized nature of the states observed in the band structure. The peculiar composition of the VBM is related to the highly conjugated π orbitals of the cations, whose shallow levels partially mix with iodine p-orbitals. As expected, the electronic band gap of 0.71 eV obtained at the PBE-SOC level of theory is largely underestimated. A more accurate estimate was obtained by performing *G*_0_*W*_0_ calculations using the electron wavefunctions obtained by Perdew–Burke–Ernzerhof (PBE)-SOC calculations, see Computational Details. By this approach, a renormalization of the electronic band gap to 2.01 eV was obtained, a value higher than the optical band gap measured at 1.6 eV, indicating the presence of large exciton effects in the material.

To clarify the nature of the optical excitations at low energies, i.e., close in energy to band-to-band transitions, the optical spectrum of the perovskite was simulated by solving the Bethe Salpeter equation (BSE) on top of the quasiparticle states calculated at the *G*_0_*W*_0_ level; the results are displayed in Figure 7, panel D. The absorption spectrum is characterized by the presence of several exciton peaks of various nature. The calculated optical excitations with the lowest energy show very small oscillator strengths and are placed at 1.46 eV, i.e., below the fundamental gap of the material (2.01 eV), indicating large exciton binding energies of 0.55 eV. These dark excitons are associated with π → π* transitions mainly involving the HOMO and LUMO orbitals of the organic cations and are slightly below the optical gap extrapolated by experiments (1.6 eV), while they quantitatively reproduce the optical feature observed in PL emission at ∼1.4 eV. The first intense exciton peak (A) is calculated at 0.21 eV below the fundamental gap, and it matches the optical feature measured by absorption spectroscopy at around 1.8 eV. Similarly to the lowest energy exciton, this excitation mainly stems from HOMO–LUMO singlet transitions of the cation (see the inset in Figure 7D). It is worth mentioning that the dark exciton solution at 1.4 eV is observed only by a fully relativistic treatment of the BSE problem, thus not restricting the spin multiplicity, while without SOC the bright exciton at 1.8 eV is the lowest energy excitation. This, coupled with the calculated low oscillator strength, highlights that the dark exciton has a mixed singlet–triplet character, and likely it can be activated in emission. Beyond 2 eV the optical spectrum is dominated by the convolution of exciton peaks derived by charge transfer processes between the inorganic moiety (I p-orbitals) and the LUMO of the cation, typical of viologen compounds. In the inset of Figure 7D, the Kohn–Sham orbitals mostly contributing to such transition in the intense peak calculated at 2.2 eV (B) are reported.

Based on the computational analysis, an interpretation of the optical features of the perovskite is provided. In absorption, π → π* transitions of the organic cation dominate the low energy portion of the optical spectrum, while at higher energies (>2 eV) these transitions overlap with charge transfer excitations between the inorganics and the organics. The optical gap extrapolated by absorption spectroscopy (1.6 eV) has an intermediate energy between the first dark exciton at 1.46 eV and the first bright exciton predicted at 1.80 eV, highlighting a generally good agreement between experiments and theory. A direct comparison between calculations and experiments, however, is complicated due to several effects, such as the presence of carrier relaxation effects resulting in a Stokes shift between absorption and emission,^47^ and temperature effects, modulating the optical gap of the perovskite through the activation of the cation vibrational modes inside the 1D channels of the perovskite. On the other hand, the bright emission at 1.4 eV is compatible with the activation of the dark exciton calculated at 1.46 eV and ascribed to the fluorescence of the cation after the relaxation of the photo-generated electron–hole pair into the LUMO and HOMO orbitals.

Finally, in order to test the suitability of AEPyPbI for thin film deposition, which is crucial for optoelectronic applications, a test was made using a spray deposition technique of a saturated solution in DMF on a microscope glass slide kept at 160 °C. The film obtained resulted to be constituted only of AEPyPbI with no decomposition products, as demonstrated by thin-film XRD (see Figure S10 in the Supporting Information). The XRD pattern of the film is superimposable with the one obtained from powder shown in Figure 3. This experiment demonstrated the possibility of obtaining films of AEPyPbI from solutions.

## Conclusions

A novel 1D hybrid iodoplumbate with a highly conjugated quaternary ammonium cation was synthesized and thoroughly characterized structurally and chemically, and its electronic properties were studied spectroscopically and using state-of-the-art quantum chemical calculations. As expected for a quaternary ammonium lead halide, the material shows high chemical and thermal stability decomposing around 300 °C, and it is water tolerant. The material shows intense PL, centered at 1.43 eV, and its integrated emission intensity is larger than that of InP in the same excitation conditions. The interesting emissive properties of the material stem from the interaction of the electronic structure of the highly conjugated organic cation with that of the inorganic sublattice. The relatively easy synthesis of the material and its optoelectronic properties make this perovskite a promising candidate for applications such as light-emitting and photovoltaic devices.

## Methods

The synthesis of AEPyPbI is described in the Supporting Information.^27^

### Thermogravimetry-Differential Thermal Analysis

TG-DTA analysis was performed with a Netzsch STA 409 PC Luxx system. A flowing Ar atmosphere (purity ≥99.9995% 85 cm^3^/min @ STP) was used for the analysis. Scan rate: 10 K/min; temperature range: 30–500 °C.

### UV–vis (Ultraviolet–Visible) Spectroscopy

The UV–vis spectrum in diffuse reflectance mode was acquired using Shimadzu (Japan) UV2600. An ISR-2600 Plus integrating sphere (BaSO_4_ reference) was used.

### FT-IR Spectroscopy

A Bruker Alpha FT-IR spectrophotometer was used in attenuated total reflection (ATR) mode using the ATR Platinum Diamond 1 accessory for the acquisition of the IR spectrum in the 400–4000 cm^–1^ wavenumber range. Resolution: 4 cm^–1^.

### Raman Spectroscopy

For Raman measurements, the excitation laser was provided by a single frequency Nd/YVO_4_ lasers (DPSS series by Lasos) emitting at 532 nm. The Raman signal was spectrally dispersed by an ACTON SP750 monochromator with a focal length of 750 mm and equipped with 300 grooves/mm grating. The signal was detected by a back-illuminated N_2_-cooled Si CCD camera (100BRX by Princeton Instruments). The laser light was filtered out by a very sharp long-pass Razor edge filter (Semrock). The spectral resolution was 2.8 cm^–1^. A 100× objective with NA = 0.9 was employed to excite and collect the light, in a backscattering configuration.

### ESI-MS

A Thermo Scientific—TSQ QUANTUM ACCESS triple quadrupole spectrometer was used for the ESI-MS analysis. AEPyPbI was dissolved in methanol for the analysis.

### TOC Analysis

The carbon content of AEPyPbI was determined using a TOC Cube system by Elementar.

### PL Spectroscopy

PL measurements were taken in the same experimental configuration used for Raman measurements. In this case, the sample was excited through a 20× objective with NA = 0.4, and the signal was spectrally dispersed by a Princeton Isoplane160 monochromator with a focal length of 200 mm and equipped with a 150 grooves/mm grating. The signal was detected by a back-illuminated N_2_-cooled Si CCD camera (100BRX by Princeton Instruments) or by an InGaAs linear array (PyLoN-IR by Princeton Instruments). To have reliable information of the PL line shape and intensity, the system response was duly taken into account. The system response was measured by using a blackbody source and by comparing the measured spectrum with the blackbody nominal one.

### Structure Determination

High-angle annular dark-field scanning transmission electron microscopy (HAADF-STEM) imaging and 3D ED were carried out with a transmission electron microscope (TEM) Zeiss Libra 120 equipped with a LaB_6_ cathode (120 kV). 3D ED was performed by keeping the TEM in STEM mode after defocusing the beam in order to have a parallel illumination on the sample, as described by Lanza et al.^48^ ED patterns were collected in Köhler parallel illumination with a beam size of about 150 nm in diameter, obtained using a 5 μm C2 condenser aperture. Data were recorded by a single-electron ASI MEDIPIX detector.^49^

The powdered sample was gently crushed and loaded directly on a carbon-coated Cu TEM grid without any solvent or sonication. 3D ED acquisitions were performed when rotating the sample around the TEM goniometer axis in steps of 1°, with a total tilt range of up to 110°. After each tilt, a diffraction pattern was acquired and the crystal position was tracked by STEM imaging. The exposure time per frame was 1 s. The camera length was 180 mm, allowing resolution in real space up to 0.7 Å. The extremely low-dose illumination allowed data to be acquired at room temperature without evidence of any sample amorphization. In order to proper integrate every reflection over the excitation error, during the experiment, the beam was precessed around the optical axis by an angle of 1°.^50^ Precession was obtained using a Nanomegas Digistar P1000 device.

3D ED data were analyzed using the *PETS2.0* software package.^51^ Structure determination of inorganic clusters was achieved by SDMs, while organic ligand molecule was added by SA, as implemented in the *SIR2014* software package.^52^ The resolution limit was set at 0.9 Å. Data were treated with a fully kinematical approximation, assuming that *I_hkl_* was proportional to |*F_hkl_*|.^2^ The model determined by SA was refined with the least-squares procedures embedded in the *SHELXL* software package.^53^ Geometrical restraints and constraints were added stepwise to check the consistency of the model. All hydrogen atoms were generated in geometrically idealized positions.

High-quality XRPD patterns were collected on a Malvern Panalytical X’Pert Pro MPD, operating in Bragg–Brentano geometry and equipped with an ultrafast RTMS X’Celerator detector. The diffraction data were acquired in the 2θ ranges of 3.5–8.5° and 8.5–90° using Cu Kα radiation Ni-filtered (λ^κα1^ = 1.54056 Å, λ^κα2^ = 1.54439 Å). A beam knife was used in the range of 3.5–8.5°, in order to suppress the main beam diffuse scattering.

Thin film X-ray diffraction measurement was performed using the thin film module equipped with a graphite monochromator of the same Malvern Panalytical X’Pert Pro MPD diffractometer. The scan was performed in parallel beam mode using a fixed incidence angle set at 1.5° in order to minimize substrate contribution.

The unit cell and structure parameters were refined with *Jana2006.*(54) The background was described by manually picked fixed points. The analyzed data collections were merged scaling the baseline of the 8.5–90° range with respect to the 3.5–8.5° baseline. Profile parameters were first obtained by Le Bail fitting and kept fixed in the Rietveld. Unfortunately, the XRPD patterns suffer from limited diffraction resolution and severe peak overlap preventing a free Rietveld refinement. The refinement on AEPyPbI was performed starting from the atomic coordinates obtained by the single-crystal 3D ED model. The organic fraction of the structural model (AEPy) was refined as semi-rigid bodies, able to rotate around their single bonds while the aromatic rings were considered rigid bodies.

### Computational Details

DFT calculations were carried out in the primitive cell of AEPyPbI by using the Quantum Espresso (QE) software package^55^ and by fixing cell parameters to the experimental values. The equilibrium positions of ions in the cell were found by performing a geometry relaxation using the PBE functional and including DFT-D3 dispersion corrections.^46,56^ Calculations were performed by using scalar relativistic norm-conserving pseudopotentials (I 5s, 5p; N and C 2s, 2p; H 1s; and Pb 6s, 6p, 5d shells explicitly included) with a cutoff on the wavefunctions of 60 Ry and an 8 × 1 × 1 *k*-point grid in the Brillouin zone (BZ). Phonon calculations were carried out at the experimental cell parameters. The ground state geometry and charge density were calculated by using the same computational setup described above but without including dispersion corrections. Phonon frequencies at the Γ point of the BZ and the relative IR spectrum were calculated at the PBE level by using DFPT,^42^ as implemented in the QE package.

The electronic structure of the perovskite was calculated at the PBE-D3 relaxed geometry by using the PBE functional and by including SOC with the use of the full relativistic version of the pseudopotentials, see Supporting Figure S8 and Table S1. The optical spectrum was calculated by solving the BS equation on top of the *G*_0_*W*_0_ calculation, for an accurate estimate of the electronic features and exciton properties of the perovskite, by using the Yambo code.^57^*G*_0_*W*_0_ calculations were carried out in the plasmon-pole approximation by using the wavefunctions provided by PBE-SOC calculations. A cutoff of 40 Ry (4 Ry) for the exchange (correlation) part of the self-energy was used, by including a total of 1000 bands (VB contains 444 electrons) in the calculation of the dielectric matrix and correlation energy. BSE calculations were performed on top of QP-corrected eigenvalues by using a cutoff of 40 Ry (4 Ry) on the exchange (screening) parts and by including 30 occupied and 30 unoccupied bands. A 10 × 1 × 1 grid of *k*-points in the BZ was used. Convergence tests on the *G*_0_*W*_0_ band gap vs computational parameters and the BSE spectrum vs the *k*-point grid are reported in the SI, see Supporting Table S2 and Figure S9.

## Supporting Information Available

Synthesis protocol, positive and negative ion ESI mass spectra, structural model and electron density maps, PL spectra, DFT calculations, and XRD pattern of a film of AEPyPbI (PDF)
