# Supplementary material for: 4,4′-(Anthracene-9,10-diylbis(ethyne-2,1-diyl))bis(1-methyl-1-pyridinium) Lead Iodide C30H22N2Pb2I6: A Highly Luminescent, Chemically and Thermally Stable One-Dimensional Hybrid Iodoplumbate
Source: Chem Mater. 2023 Feb 15;35(4):1818–26. doi: 10.1021/acs.chemmater.2c03798 (PMC9979375; doi:10.1021/acs.chemmater.2c03798)
Supplement: Supplementary file 1 — cm2c03798_si_001.pdf [file cm2c03798_si_001.pdf]

Supporting Information for

4,4'-(anthracene-9,10-diylbis(ethyne-2,1-diyl))bis(1-methyl-1-pyridinium) lead iodide $C_{30}H_{22}N_2Pb_2I_6$: a highly luminescent, chemically and thermally stable 1D hybrid iodoplumbate

Lorenza Romagnoli[1], Andrea D'Annibale[1], Elena Blundo[2*], Atanu Patra[2], Antonio Polimeni[2], Daniele Meggiolaro[3*], Iryna Andrusenko[4], Danilo Marchetti[4,5], Mauro Gemmi[4*], Alessandro Latini[1*]

[1] *Dipartimento di Chimica, Sapienza Università di Roma, Piazzale Aldo Moro 5, 00185 Roma, Italy*

[2] *Dipartimento di Fisica, Sapienza Università di Roma, Piazzale Aldo Moro 5, 00185 Roma, Italy*

[3] *Computational Laboratory for Hybrid/Organic Photovoltaics (CLHYO) Istituto CNR di Scienze e Tecnologie Chimiche "Giulio Natta" (CNR-SCITEC) Via Elce di Sotto 8, 06123 Perugia, Italy*

[4] *Electron Crystallography, Center for Materials Interfaces, Istituto Italiano di Tecnologia, Viale Rinaldo Piaggio 34, 56025 Pontedera, Italy.*

[5] *Department of Chemistry, Life Sciences and Environmental Sustainability, University of Parma, Parco Area delle Scienze 17/A, 43124 Parma (PR), Italy*

*corresponding authors

Synthesis of $AEPyPb_2I_6$:

$$AEPyI_2 + 2PbI_2 + 3NaI \rightarrow AEPyPb_2I_6$$

In a glass vial, 38 mg (0.0572 mmol) of **$AEPyI_2$**[27] are dissolved in 3 mL of H_2O; in another vial, 53 mg (0.115 mmol) of PbI_2 and 26 mg (0.173 mmol) are dissolved in 9 mL of acetone. The first solution, containing the organic salt, is then slowly added, dropwise, to the second: instantaneously, a dark red-brown solid starts to form. After addition is complete, the mixture is vigorously shaken, then it is left undisturbed for 24 hours, during which the precipitate changes color to dark violet-black. After that, the dark solid is filtered under vacuum and washed with several portions of a 3:1 acetone/water mixture, then it is dried for 2 hours under suction, to yield 91 mg of crude product. The solid is subsequently dissolved in 75 mL of DMF, the dark red solution is filtered over a 0.45 μm PTFE syringe filter and exposed to CH_2Cl_2 vapors for 72 hours in a closed container. After vacuum filtration, the product is washed with multiple portions of CH_2Cl_2 and allowed to dry for 1 hour under suction, to yield 60 mg of pure **$AEPyPb_2I_6$** as black crystals (66%). Samples for structure determination by electron diffraction were grown by exposing saturated solutions of **$AEPyPb_2I_6$** in DMF in silanized glass vials, covered by aluminum foil with a single small hole in it, to CH_2Cl_2 vapors, in a closed vessel, for 1 week at 22 °C. Elemental analysis, calculated: C 22.70%, H 1.40%, N 1.77%; found C 23.99% H 1.45%, N 1.87%.

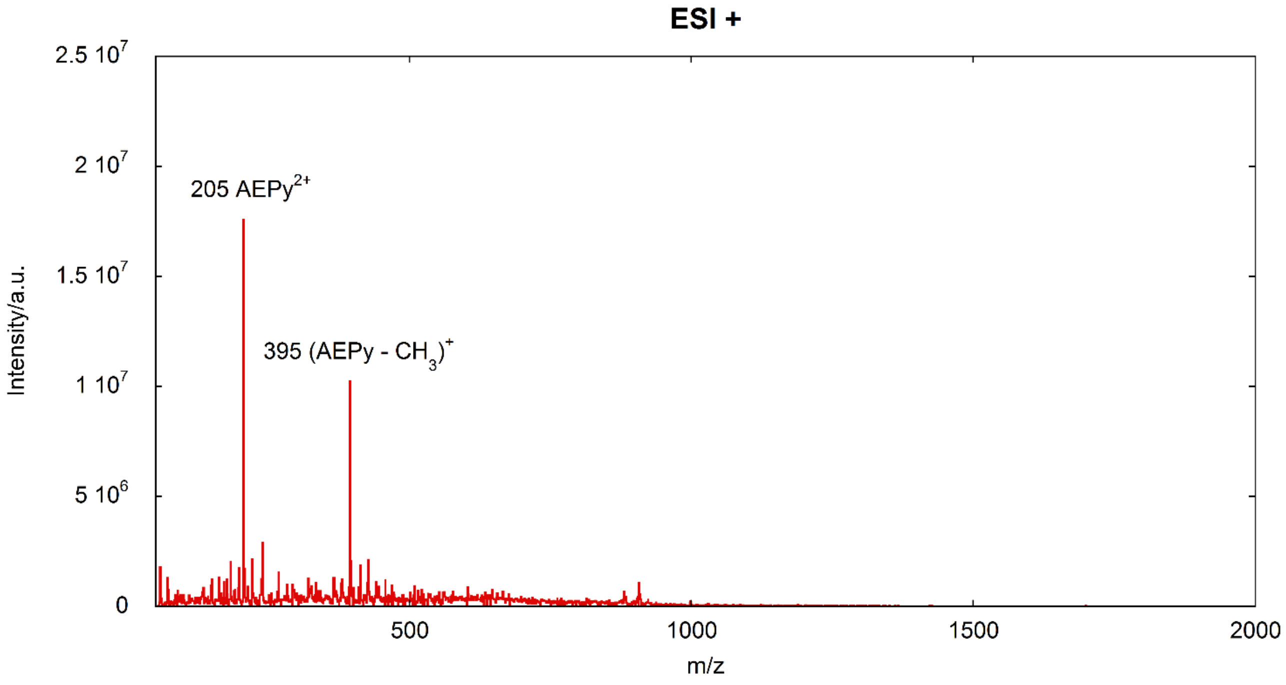

Figure S1. Positive ions ESI mass spectrum of AEPyPI.

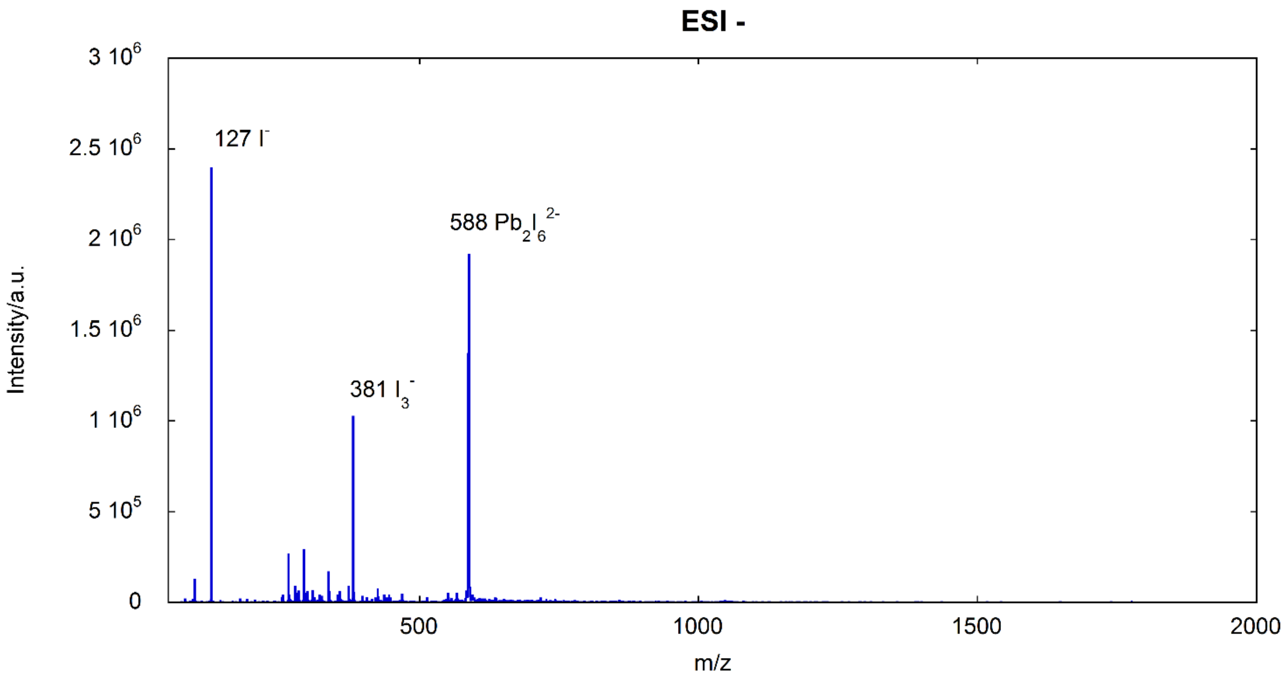

Figure S2. Negative ions ESI mass spectrum of AEPyPI.

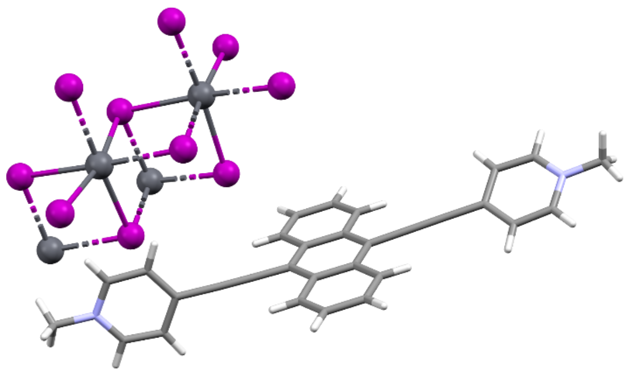

Figure S3. Asymmetric unit of $AEPyPb_2I_6$ structural model. Color Code: Pb (dark-grey), I (purple), N (blue), C (light-grey), H (white).

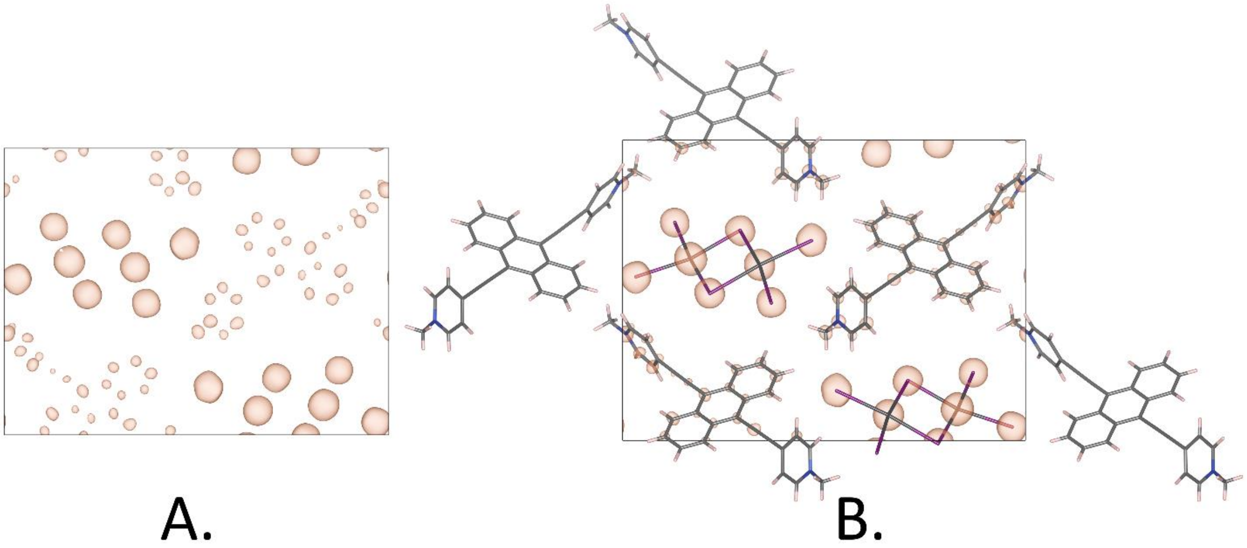

Figure S4. (A) Electron density map representation of $AEPyPb_2I_6$ calculated from XRPD data. (B) Superposition of electron density map and structural model of $AEPyPb_2I_6$.

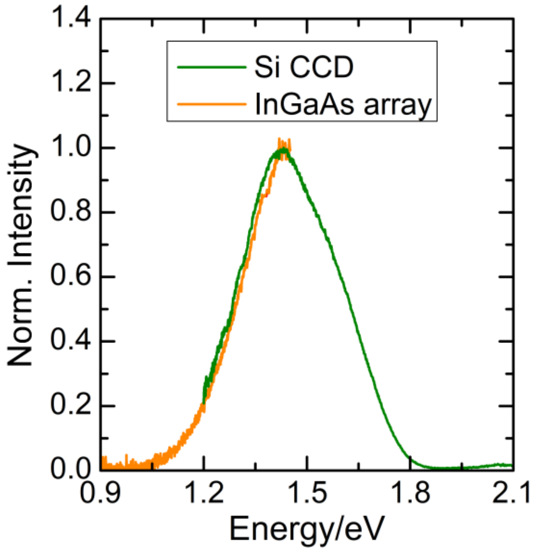

Figure S5. PL spectra of AEPyPbI acquired with a Si-CCD and with an InGaAs array. Both spectra were normalized by the system response.

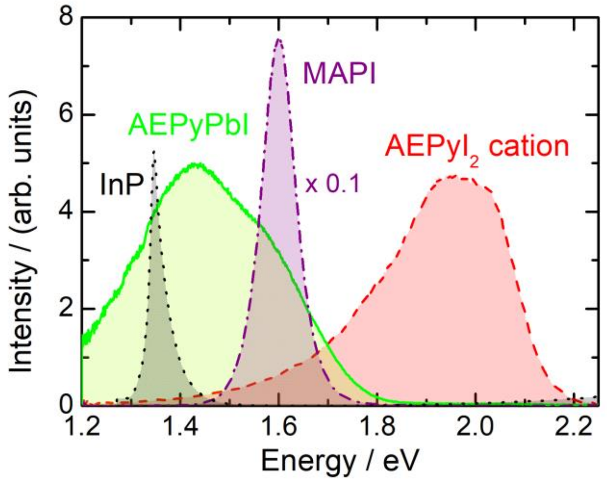

Figure S6. PL spectrum of AEPyPbI as compared to that of the $AEPyI_2$ cation and to those of an InP epilayer, and a $CH_3NH_3PbI_3$ (MAPI) powder perovskite. The laser was focused on the sample trough a 20× objective with NA = 0.4. To get further quantitative information on the PL efficiency, we measured the PL external quantum yield (PLQY) with the method proposed in ref. [Amani et al., Science 350, 1065 (2015)]. As a reference, besides measuring the PLQY of AEPyPbI, we also measured that of MAPI. The PLQYs that we measured for AEPyPbI and for MAPI are 17 % and 25 %, respectively, under laser excitation with power density of about 100 kW/m^2. This power density is much higher than that typically used in integrating spheres, due to the fact that the method employed in this work is more sensitive to stray light and high powers are thus needed to make the background signal negligible. The measured PLQY of MAPI (25 %) is a bit larger than that reported in previous works, ~5-15 %[42-44]. In those works, however, power densities lower by a factor of 100 were employed, and the measurements were performed on thin films deposited on substrates rather than on powders like in our case. In thin films, losses at the film/substrate interface are introduced, leading to a reduced PLQY[42]. These factors possibly justify the larger PLQY measured herein for MAPI. Indeed, the PLQY of AEPyPbI is only a bit lower (17 %) than that of MAPI, suggesting a good PL efficiency for this perovskite.

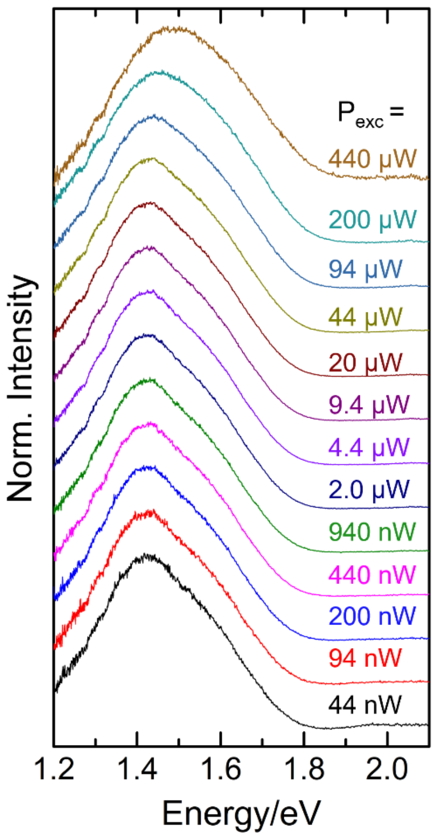

Figure S7. PL spectra of AEPyPbI as a function of the excitation laser power. The laser was focused on the sample trough a 20× objective with NA = 0.4. The spectra are normalized and stacked by y-offset for ease of comparison. No significant lineshape variation is observed up to 200 μW, which testifies the robustness of this perovskite against photoexcitation. At higher powers, a broadening is observed, followed by a quenching of the PL signal.

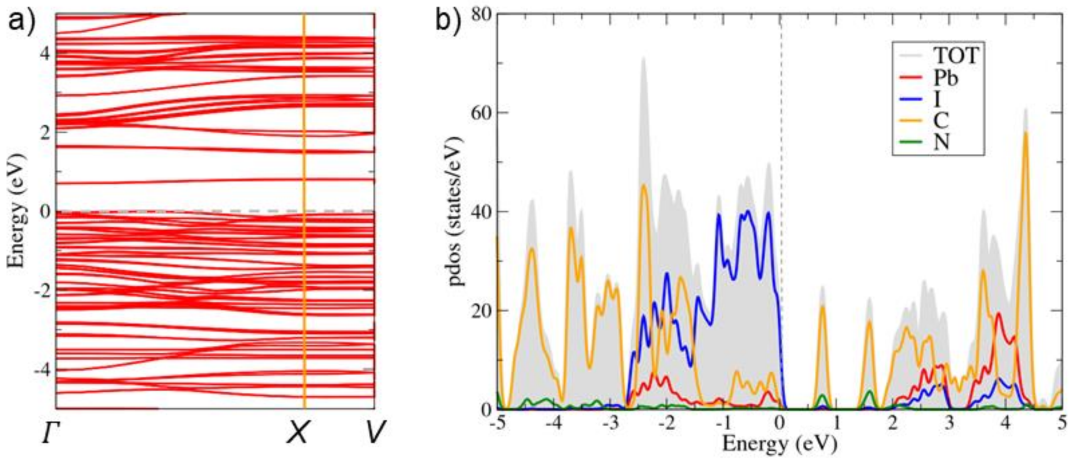

Figure S8. a) Electronic band structure and b) projected density of states (PDOS) of the AEPyPbI perovskite calculated by using the PBE functional and by including spin-orbit coupling (SOC).

Table S1. Experimental vs optimized cell parameters of the AEPyPbI perovskite obtained by performing a full relaxation of cell and ions with the PBE functional and by including DFT-D3 dispersions. Calculated band gaps at the PBE level for the different structures.

	Cell parameters	Eg (eV) - PBE
PBE-D3, relaxed ions / experimental cell	a=4.700 Å, b=23.000 Å, c=17.800 Å α= 90.0°, β=105.0°, ɣ=90.0°	0.78
PBE-D3, relaxed ions and cell	a=4.661 Å, b=22.855 Å, c=17.764 Å α= 90.0°, β=105.6°, ɣ=90.0°	0.75

Table S2. Convergence of the band gap vs computational parameters in G_0W_0 calculations. Convergence on the number of bands and cutoff on exchange (EXXRLvcs) and dielectric matrix (NGsBlkXd) have been carried out in the plasmon-pole approximation, without including SOC to reduce computational effort. In all cases the same number of bands have been used to calculate dielectric matrix and correlation energy.

**Computational setup**	**G_0W_0 band gap (eV)**
(kp grid, EXXRLvcs, NGsBlkXd, number of bands)	
4x1x1, 20 Ry, 2Ry, 400	1.94
4x1x1, 30 Ry, 3 Ry, 400	1.98
4x1x1, 40 Ry, 4 Ry, 400	2.01
4x1x1, 20 Ry, 2Ry, 400	1.94
4x1x1, 20 Ry, 2 Ry, 600	1.88
4x1x1, 20 Ry, 2 Ry, 800	1.86
8x1x1, 40 Ry, 4 Ry, 800	1.97
10x1x1, 40 Ry, 4 Ry, 800	2.01
12x1x1, 40 Ry, 4 Ry, 800	2.05

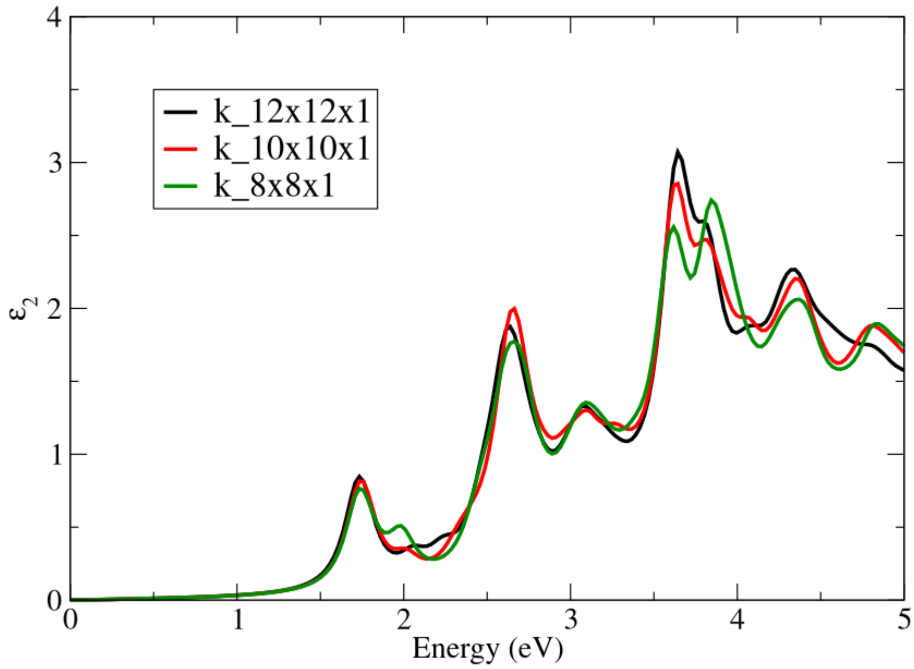

Figure S9. Convergence of the calculated BSE spectra vs the k-points in the BZ. BSE calculations have been performed on top of G_0W_0 corrected eigenvalues, without including SOC to speed up convergence tests. The number of bands for the screening term has been set to 800 (222 occupied bands), cutoff of 40 Ry (4 Ry) have been used for the exchange (screening) in the G_0W_0 – ppa and BSE calculations. The spectra have been calculated as the optical response to electric field in the (1,1,1) direction in space by including 25 occupied and 25 unoccupied bands.

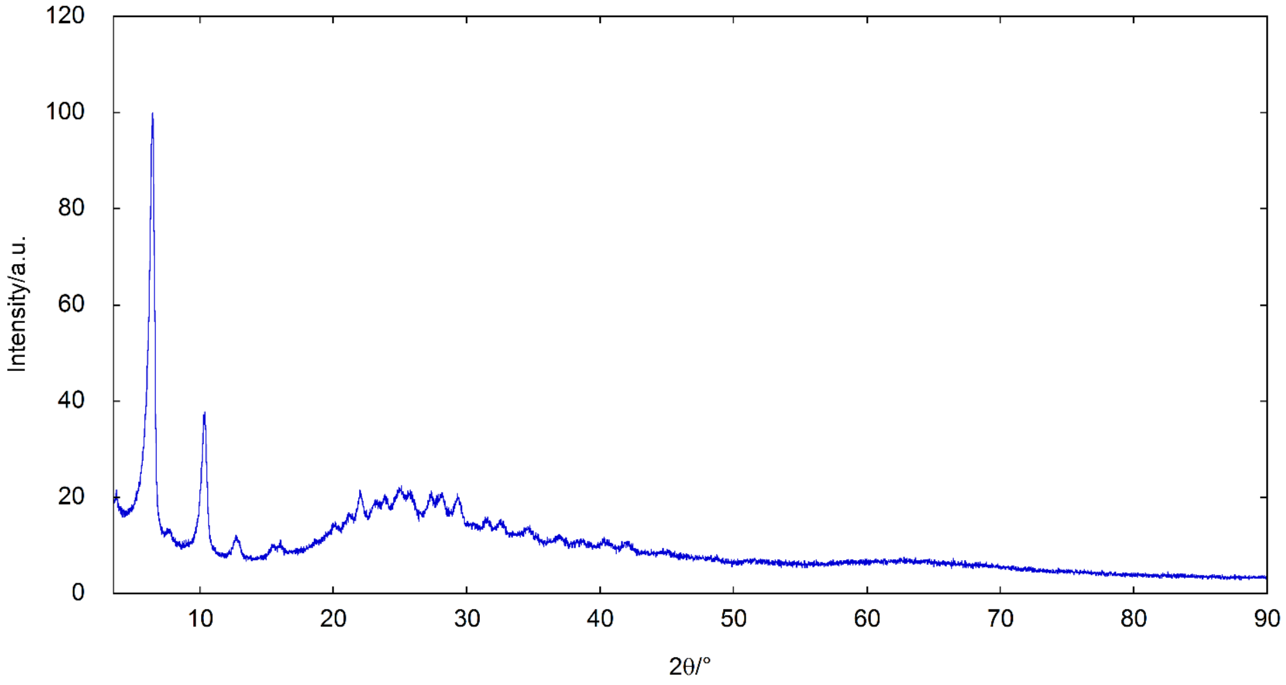

Figure S10. XRD pattern of a film of AEPyPbI obtained by spray deposition on a glass slide at 160 °C.
